# Circadian variation and responsiveness of hydration biomarkers to changes in daily water intake

**DOI:** 10.1007/s00421-013-2649-0

**Published:** 2013-04-23

**Authors:** Erica Perrier, Agnès Demazières, Nicolas Girard, Nathalie Pross, Dominique Osbild, Deborah Metzger, Isabelle Guelinckx, Alexis Klein

**Affiliations:** 1 Forenap, 27 rue du 4éme RSM, 68250 Rouffach, France; 2 Danone Research, RD 128, 91767 Palaiseau, France

**Keywords:** Water, Fluid intake, Saliva, Urine, Osmolality, Hydration, Circadian

## Abstract

Biomarkers of hydration change in response to acute dehydration; however, their responsiveness to changes in fluid intake volume, without exercise or heat exposure, has not been adequately described. Moreover, patterns of circadian variation in hydration biomarkers have not been established. The study aims were to (1) assess the response of hydration biomarkers to changes in daily water intake; and (2) evaluate circadian variation in urinary and salivary biomarkers. Fifty-two adults (24.8 ± 3.1 years; 22.3 ± 1.6 kg/m^2^; 79 % female), grouped based on habitual fluid intake (low drinkers, *n* = 30, <1.2 L/day; high drinkers, *n* = 22, >2.0 L/day), completed a 5-day inpatient crossover trial. On days 1 and 2, low drinkers received 1.0 L/day of water while high drinkers received 2.5 L/day. On days 3 through 5, intake was reversed between groups. Plasma and saliva osmolality were assessed daily at predetermined times, and all urine produced over 24 h was collected in timed intervals. ANOVA with intake (1.0 vs. 2.5 L/day), day, and time revealed that (1) urine concentration (osmolality, specific gravity, color) and volume, but not plasma nor saliva osmolality, responded to changes in water intake; (2) urinary hydration biomarkers and saliva osmolality vary as a function of the time of day; and (3) urine osmolality measured in samples collected during the afternoon most closely reflects the corresponding 24 h value. Overall, urinary hydration biomarkers are responsive to changes in water intake, and stabilize within 24 h of modifying intake volume. Moreover, short afternoon urine collections may be able to replace 24 h collections for more convenience in hydration assessment.

## Introduction

Water is an essential nutrient and is the main component of the human body, comprising 73 % of lean body mass (Peronnet et al. 2012), and approximately 50–60 % of total adult body weight (Watson et al. 1980). At the population level, recommendations for adequate total water intake have been established by many regional and national health authorities, based largely on median water intakes from national population surveys (EFSA 2011; IOM 2004) and without solid physiological evidence linking total water intake to hydration biomarkers in urine, saliva, or blood. Thus, it remains difficult to accurately establish individual water needs, which are influenced by factors including body size, activity level, dietary habits, metabolic rate, climate, and urine concentrating capacity. The health implications of adequate daily intake have recently been highlighted. Low daily fluid intake increases the risk of chronic kidney disease (Strippoli et al. 2011) and lithiasis (Curhan et al. 2004; Sorensen et al. 2012); low water intake may also play a role in the development of hyperglycemia (Roussel et al. 2011). Urinary biomarkers of hydration have been shown to vary as a function of fluid intake (Armstrong et al. 2010, 2012; Perrier et al. 2012); moreover, low urine volume and high urine concentration have been, respectively, associated with an increased risk of chronic kidney disease (Clark et al. 2011) and lithiasis (Hennequin et al. 1995). Thus, given the links between low intake, disease risk, and urinary biomarkers, a precision of adequate intake that takes physiological indicators of hydration into account would represent an improvement in the accuracy of water intake recommendations for individuals.

Numerous biomarkers have been considered as indicators of hydration status, including changes in body weight, as well as plasma and urinary indices (Armstrong 2007; Armstrong et al. 2010; Cheuvront et al. 2011; Kavouras 2002). However, the relative accuracy and usefulness of any single biomarker appear to be dependent on the context in which dehydration is achieved, whether induced by exercise, temperature, or a combination of both stressors (Armstrong et al. 1998; Francesconi et al. 1985), or by fluid restriction (Oliver et al. 2008; Pross et al. 2012). Little is known about the expression of these hydration biomarkers in ‘average’ living conditions, when water losses are moderate and intake is the major determinant of water balance. Differences in urinary biomarkers of hydration, but not in plasma osmolality, have been reported between individuals who habitually consume low versus high daily fluid volumes (Perrier et al. 2012). However, understanding the dynamic responsiveness of hydration biomarkers to changes in fluid intake is essential. A pair of recent studies have reported inverse relationships between habitual fluid intake and measures of urine volume and concentration in healthy men and women (Armstrong et al. 2010, 2012). The results reported significant differences between the concentration of first morning and 24 h urine samples, and therefore the possibility that urinary biomarkers are influenced by circadian fluctuations that have not yet been well characterized.

In addition to hydration biomarkers in urine and plasma, recent studies have explored the potential of saliva osmolality as a biomarker of hydration. Conceptually, saliva osmolality is attractive, as it is non-invasive and easier to sample relative to blood or urine; however, its sensitivity is not clearly established. Saliva osmolality has been reported to be as sensitive to acute exercise-induced dehydration as urine osmolality (Walsh et al. 2004), while its sensitivity as a hydration marker has also been questioned due to substantial intra- and inter-individual variability (Taylor et al. 2012). Moreover, the reliability of the measurement may be impacted by oral artifact such a water mouth rinse (Ely et al. 2011): thus, saliva osmolality may also be influenced by regular daily activities such as eating and drinking. As with urinary indices, the ability of saliva osmolality to track changes in hydration status has been evaluated in situations of acute dehydration, and little is known about the variability of saliva osmolality in the general population, where water balance is largely determined by intake and not loss. Moreover, the possibility that saliva osmolality undergoes regular daily fluctuations has not been explored.

The purpose of this study was to satisfy two specific aims. Our first aim was to assess the response of hydration biomarkers to changes in water intake. To satisfy this aim, hydration biomarkers in urine, saliva and blood were assessed before and after an increase or decrease in the volume of plain water ingested daily. Our second aim was to establish the presence of circadian variation in urinary and salivary hydration biomarkers.

## Methods

### Experimental approach and subjects

This prospective study was performed on two non-randomized, parallel groups, who underwent a crossover intervention. The study was conducted at a single investigating center according to the ethical principles stated in the revised version of the Declaration of Helsinki and approved by an Independent Ethics Committee. Fifty-two young healthy non-smoking adults (age 24.8 ± 3.1 years, BMI 22.3 ± 1.6 kg/m^2^, 79 % female) participated in the study after giving their written informed consent. Inclusion criteria included the use of monophasic contraception (females) and the ability to avoid moderate and vigorous physical activity throughout the study period. Exclusion criteria included any disease or medication that may impact hydration status or water balance, such as chronic kidney disease or use of diuretics. Prior to inclusion, subjects’ habitual fluid intake was self-reported using an e-diary (Neometis^®^-24WQ-Waters questionnaire) during 3 consecutive days, and this information was used to allocate subjects to low drinker (*n* = 30, <1.2 L/day, 63 % female) or high drinker (*n* = 22, 2–4 L/day, 100 % female) groups.

### Study design

**Fig. 1:**
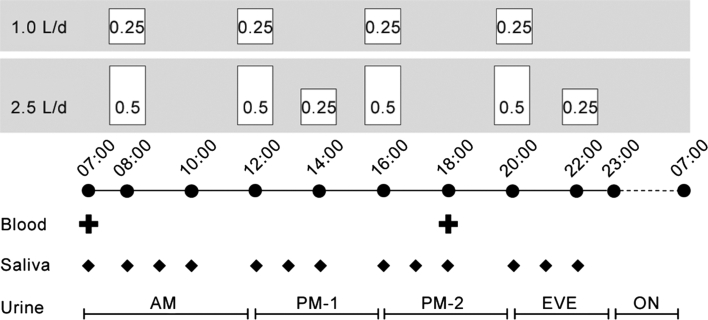
Daily schedule of water intake and hydration biomarker collections. Standardized meals were provided at 0800, 1200, and 2000 hours, with a snack at 1600 hours. Spot collections for blood (2 samples) and saliva (13 samples) were performed at predetermined times. All urine produced during the 24 h period was collected in five ‘short collection’ intervals, corresponding to morning (AM), early afternoon (PM-1); late afternoon (PM-2); evening (EVE); and overnight (ON)

Participants arrived at the study center in the afternoon, and would remain at the study center until the end of the intervention. Beginning at 0700 hours the next morning, subjects began a 2-day baseline period (baseline: D1 and D2) during which they were provided with 1.0 L/day (low drinkers) or 2.5 L/day (high drinkers) of water (Volvic^®^, France). Next, for 3 days following baseline, fluid intakes between groups were reversed (intervention: D3, D4, and D5). In both the 1.0 and 2.5 L/day intake conditions, water was provided in pre-measured volumes according to a set daily schedule (Fig. 1), meals and snacks were timed and standardized on all days, and physical activity was restricted to sedentary activities. As a consequence of the controlled timing of daily water intake, the 2-day baseline was followed by a 3-day intervention, to ensure two full 24 h periods (D4–D5) at the increased (for low drinkers) or decreased (for high drinkers) level of water intake.

### Biomarker sampling: daily schedule

Hydration biomarkers in blood and saliva were assessed repeatedly each day according to a predetermined schedule (Fig. 1). Over each 24 h period, all urine produced was collected in five separate containers, termed ‘short collections’: morning (0700–1200 hours), early afternoon (1200–1600 hours); late afternoon (1600–2000 hours); evening (2000–2300 hours); and overnight (2300–0700 hours). All urine produced during each collection interval was collected in the single container, regardless of the number of voids in a collection interval. The bladder was emptied prior to beginning each new short collection; thus, each short collection represented the total volume of urine produced during a determined time interval. Venous blood samples were drawn at 0700 hours (fasting) and 1800 hours daily. A total of 13 saliva samples were obtained between 0700 and 2200 hours, with a minimum elapsed time of 30 min between eating or drinking and the collection of the sample.

#### Urine

Urine analyses were carried out on each short collection separately, and were subsequently repeated on pooled 24 h samples. Urine osmolality (UOsm) was measured using a freezing point depression osmometer (Model 3320, Advanced Instruments Inc., Norwood, MA, USA). Specific gravity (USG) was measured with a digital USG pen refractometer (Atago Ltd.). Urine color (UCol) was determined via the eight-point urine color chart developed by Armstrong et al. (1994, 1998), and volume (UVol) was measured to the nearest milliliter. In addition, to facilitate the comparison of urine production rate between collection intervals of different durations (AM: 5 h; PM-1 and PM-2: 4 h each; EVE: 3 h; ON: 8 h), UVol produced during each collection interval was divided by the duration of the collection interval to produce a relative UVol measure in mL/h (UVol_h_).

#### Plasma

Venous blood samples (5 mL) were collected in non-heparinized tubes in the morning (0700 hours) and evening (1800 hours) for determination of plasma osmolality (POsm), which was measured via freezing point osmometer in fresh morning and evening samples.

#### Saliva

Unstimulated whole saliva samples (≥200 μL) for determination of saliva osmolality (SOsm) were collected. The participant sat quietly for 2 min to allow saliva to passively collect under the tongue, with minimal orofacial movements. The collected saliva sample was then expectorated into a collection tube, and osmolality was determined on the fresh sample using the freezing point osmometer.

### Statistical analysis

Analyses were performed using SAS (v.9.1.3; Cary, NC, USA). All parameters were checked for sex differences; none were found, and therefore data for men and women in the low drinkers group were collapsed. Low and high drinkers were analyzed separately using ANOVA with intake (1.0 vs. 2.5 L/day), day (D1–D5, nested within intake), time (for urine, this corresponds to the short collection intervals), and intake by time interaction. All tests were two-sided with an alpha of 0.05. Main effects of intake and day were examined to determine the responsiveness of hydration biomarkers to the change in water intake; while the circadian variation of urine and saliva biomarkers was evaluated based on the effect of time as well as the intake × time interaction.

In order to evaluate the potential of each short urine collection period to provide a reasonable estimate of values measured on the full 24 h sample, urine osmolality measured on each short collection was compared to the corresponding 24 h value. Osmolality was the measure selected for this analysis because its physiological range (50–1,200 mOsm/kg) is quite broad (IOM 2004), providing a higher degree of measurement resolution than urine specific gravity or color. Taking into account the physiological range as well as 24 h UOsm values previously observed at different levels of ad libitum fluid intake (Perrier et al. 2012), a difference of ±50 mOsm/kg between the short collection sample and the 24 h collection was considered to be a reasonable threshold for accuracy in estimating 24 h osmolality from a short collection. Thus, to evaluate whether all short urine collection periods were equally suitable for estimating 24 h UOsm, a Chi-square statistic was used to compare the number of UOsm values that were within ±50 mOsm/kg of the 24 h value in each of the short collection periods.

## Results

POsm and SOam were not different across intake levels in either low or high drinkers (POsm: *p* = 0.82 and 0.16 for low and high, respectively; SOsm: *p* = 0.09 in both groups). In contrast, all urine biomarkers in both groups changed significantly in response to a change in water intake (Table 1). In low drinkers, 24 h urine concentration (UOsm, USG, and UCol) decreased significantly, while UVol increased (all *p* < 0.001). An inverse response was observed in high drinkers who reduced their water intake, where 24 h urine volume was significantly decreased and UOsm, USG, and UCol were significantly increased (all *p* < 0.001). In some, but not all, urinary hydration biomarkers, values on D3 were significantly different from baseline (D1–D2), but also different from values on D4–D5. In low drinkers on D3, UOsm was higher compared to D4 and D5 (*p* ≤ 0.003), USG was higher compared to D5 (*p* = 0.01), and UCol was higher compared to D4 and D5 (*p* < 0.001). In high drinkers on D3, UOsm, USG, and UVol were higher than on D4 or D5 (all *p* ≤ 0.02).

**Table 1:** Daily values (mean ± SD) for 24 h urine biomarkers and plasma osmolality during baseline fluid consumption (D1–D2, low drinkers 1.0 L/day, high drinkers 2.5 L/day) and during the intervention period (D3–D5, low drinkers 2.5 L/day, high drinkers 1.0 L/day)

	Baseline		Intervention			*p* value
	D1	D2	D3	D4	D5	Baseline vs. intervention
24 h UOsm (mOsm/kg)						
Low	807 ± 209	875 ± 203	409 ± 97*^,†^	377 ± 103	389 ± 73	<0.001
High	334 ± 68	331 ± 46	652 ± 105*^,†^	761 ± 147	748 ± 177	<0.001
24 h USG						
Low	1.021 ± 0.005	1.022 ± 0.005	1.011 ± 0.003^†^	1.010 ± 0.003	1.010 ± 0.002	<0.001
High	1.010 ± 0.003	1.009 ± 0.001	1.018 ± 0.003*^,†^	1.020 ± 0.004	1.019 ± 0.005	<0.001
24 h UCol						
Low	5.5 ± 1.2	5.4 ± 1.2	3.3 ± 1.2*^,†^	2.8 ± 0.9	2.4 ± 0.8	<0.001
High	2.6 ± 0.7	2.5 ± 0.7	4.8 ± 1.1	5.5 ± 1.1	4.2 ± 1.4	<0.001
24 h UVol (ml)						
Low	1,080 ± 364	971 ± 288	2,246 ± 554	2,414 ± 453	2,326 ± 396	<0.001
High	2,406 ± 537	2,481 ± 273	1,238 ± 238*^,†^	1,061 ± 220	1,031 ± 294	<0.001
0700 hours POsm (mOsm/kg)						
Low	292 ± 8	291 ± 5	292 ± 5	290 ± 6	291 ± 5	NS
High	289 ± 6	287 ± 4	288 ± 9	289 ± 3	290 ± 5	NS
1800 hours POsm (mOsm/kg)						
Low	291 ± 5	290 ± 5	290 ± 5	291 ± 5	293 ± 7	NS
High	290 ± 8	287 ± 4	288 ± 3	289 ± 6	291 ± 5	NS
SOsm (mOsm/kg)						
Low	72 ± 20	77 ± 27	71 ± 21	73 ± 27	71 ± 23	NS
High	68 ± 22	70 ± 20	71 ± 20	74 ± 25	70 ± 17	NS

*UOsm* urine osmolality, *USG* urine specific gravity, *UCol* urine color, *UVol* urine volume, *POsm* plasma osmolality, *SOsm* saliva osmolality, *Low* low drinkers, *High* high drinkers

* D3 significantly different than D4

^†^D3 significantly different than D5

**Fig. 2:**
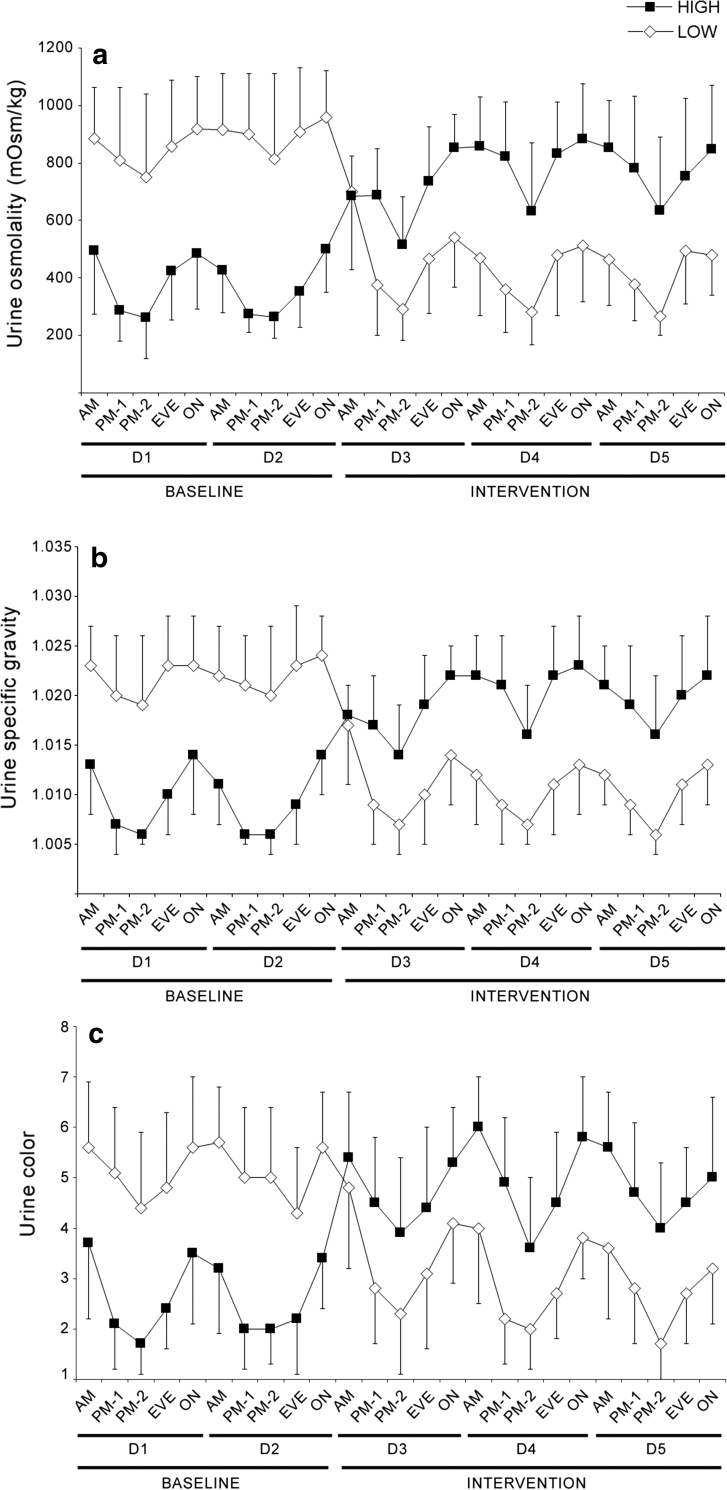
Urine osmolality (**a**), specific gravity (**b**), and color (**c**) measured from short urine collections during baseline (D1–D2) and intervention (D3–D5). Significant main effects of time were present for all three measures (*p* < 0.001 in both groups). Urinary hydration biomarkers were significantly higher during the overnight and morning collection intervals, compared with early and late afternoon. *LOW* low drinkers, *HIGH* high drinkers, *AM* morning, *PM-1* early afternoon, *PM-2* late afternoon, *EVE* evening, *ON* overnight

In both groups, under both water intake conditions, all urinary hydration biomarkers (Fig. 2) were subject to circadian fluctuations (main effect of time: all *p* < 0.001). Urine produced during the overnight and morning collections was significantly more concentrated compared with the early and late afternoon collections, regardless of group or water intake condition (all *p* < 0.05). In UOsm, USG, and UCol, the lowest daily value was typically in the late afternoon, and peak concentration was measured during the overnight or morning collections.

Urine osmolality measured on samples collected in the early or late afternoon was far more likely to accurately reflect 24 h urine osmolality, compared to morning, evening, or overnight collections. UOsm values obtained from the late afternoon collection (1600–2000 hours) were the most likely to agree with the 24 h value, with 87 % (173 of 198 measures) of values falling within 50 mOsm/kg of the corresponding 24 h value (*χ* ^2^ = 12.4, *p* = 0.004). In descending order of agreement with 24 h UOsm were early afternoon (75 %), evening (48 %), morning (46 %) and overnight (37 %).

**Fig. 3:**
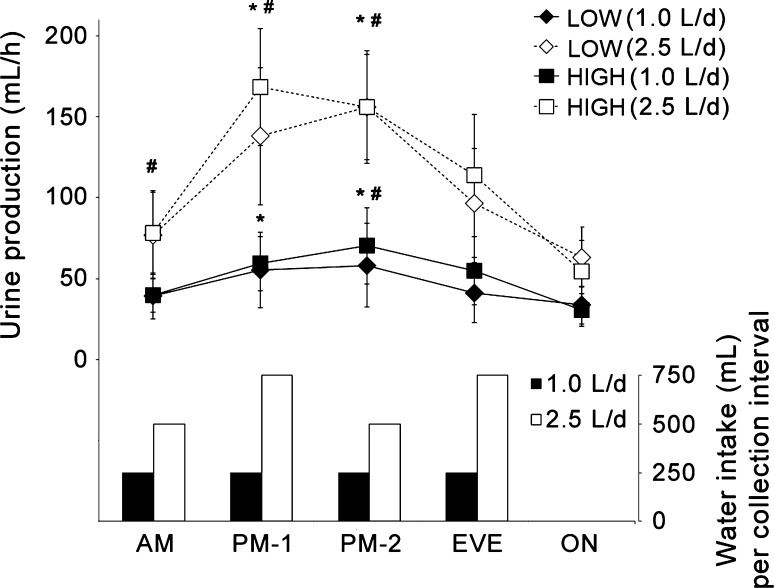
Urine production (mL/h) and volume of water (mL) ingested during the five daily urine collection intervals. *LOW* low drinkers, *HIGH* high drinkers, *AM* morning, *PM-1* early afternoon, *PM-2* late afternoon, *EVE* evening, *ON* overnight. *Significantly different from AM (*p* < 0.05), ^#^significantly different from EVE (*p* < 0.05)

The lowest urine production rate (UVol_h_; Fig. 3) occurred overnight, during which no water was provided. Significant differences were also observed between the daytime collections. In the 1.0 L/day intake condition, in both groups, urine production during early and late afternoon was higher than during morning, and late afternoon was also significantly higher than evening (all *p* ≤ 0.01). In the 2.5 L/day intake condition, early and late afternoon urine production was significantly higher than morning and evening intervals, and morning was also significantly lower than evening (all *p* ≤ 0.02).

**Fig. 4:**
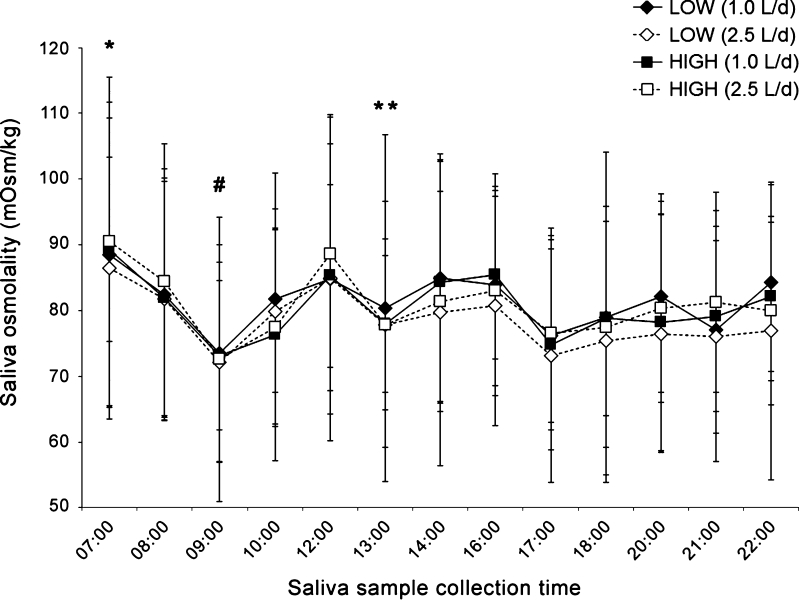
Daily fluctuations in SOsm. In both groups and under both intake conditions, SOsm was highest at 0700 hours, with significant drops in SOsm in both groups after breakfast and lunch. *LOW* low drinkers, *HIGH* high drinkers. *Higher (*p* < 0.05) compared to all other measured timepoints, with the exception of 1200 hours (low drinkers). ^#^Lower (*p* < 0.05) than 0800 hours (both groups) and 1000 hours (low drinkers). **Lower (*p* < 0.05) than 1200 and 1400 hours

First morning SOsm (0700 hours) was significantly higher than every other measured time point (Fig. 4), with the exception of one time point (1200 hours) in the low drinkers group. Moreover, noticeable and statistically significant drops were apparent in SOsm samples taken within 1 h after eating breakfast (0800–0830 hours) or lunch (1200–1230 hours; i.e., samples obtained at 0900 and 1300 hours). In low drinkers only, a drop in SOsm was also seen 1 h following the afternoon snack (1700 hours). The mean decrease (95 % CI; *p* value) after breakfast was 12 (5–20; *p* ≤ 0.001) mOsm/kg in both groups. The mean decrease (95 % CI) after lunch was 11 (4–17; *p* = 0.003) and 9 (2–16; *p* = 0.02) mOsm/kg in low and high drinkers, respectively.

## Discussion

The monitoring of hydration biomarkers is useful in establishing an adequate daily water intake volume that is adapted to the needs of the individual. In the current study, we assessed the responsiveness of hydration biomarkers in urine, blood, and saliva to a change in water intake. Using carefully controlled water intake and a crossover intervention, the results demonstrate that measures of urine concentration (osmolality, specific gravity, and color) and urine volume respond rapidly to changes in water intake, and stabilize within 24 h of modifying intake volume. With respect to urinary hydration biomarkers, the principal findings of this study were that (1) 24 h urine concentration and volume change rapidly in response to a change in water intake; (2) circadian variation influences urine concentration and volume; and (3) for measurement of urine osmolality, samples taken in the afternoon appear to best reflect the 24 h collection. With respect to saliva osmolality, the main finding was that without acute water loss due to exercise or heat exposure, saliva osmolality is not different between low and high drinkers, varies widely between individuals, and is influenced temporarily by food and beverage ingestion. Finally, the results suggest that plasma osmolality is not responsive to changes in daily water intake.

### Responsiveness of urinary hydration biomarkers to changes in daily water intake

Upon changing water intake volume, urine volume and concentration responded quickly and stabilized within 24 h. Values for urinary hydration biomarkers essentially matched the baseline values observed in the opposite group, 24 h after initiating the water volume intervention. It is noteworthy that despite tight controls over the timing and volume of water intake, there was surprising interindividual variation in urine output and concentration. At baseline, in those consuming 1.0 L/day of water, 24 h urine volume ranged from 350 to 1,483 mL, with osmolality ranging from 435 to 1,123 mOsm/kg. The range is striking, as it represents a fourfold disparity in urine output and a nearly threefold gap in osmolality, despite standardized food and water intake and a restrictive range in participant body size. Likewise, those consuming 2.5 L/day of water also produced widely different volumes of urine (1,677–3,005 mL/day, osmolality between 229 and 440 mOsm/kg). Nonetheless, previous studies do suggest a direct relationship between fluid intake and 24 h urine volume (Armstrong et al. 2010, 2012). In the current study, the range of urine volume and osmolality observed despite the restrictive study conditions speaks to a strong individuality of intrinsic regulation of body water.

### Circadian variation in urinary hydration biomarkers

Separating each 24 h period into short urine collection intervals revealed daily fluctuations in urine production. Urine volume was lower overnight, throughout the morning, and in the evening before going to sleep, with a significant increase in production in the afternoon. This fluctuation could not be explained by the timing of water intake, because intake was spaced relatively evenly through the morning, afternoon, and evening hours. This was especially apparent on the days of high (2.5 L/day) water consumption, where 750 mL was consumed during and after supper, between 2000 and 2200 hours. Despite a substantial water intake late in the evening, urine production rate dropped during the evening interval, and remained low overnight and throughout the morning, despite an additional 500 mL consumed with breakfast. This in particular has clinical relevance because urine concentration varies inversely with urine volume, and therefore concentration measures will vary in part based on time of day, independent of fluid intake. The circadian pattern of arginine vasopressin release that restricts night time urine production is documented (George et al. 1975), and the discrepancy between first morning urine and 24 h concentration has already been noted (Armstrong et al. 2010). These results go further to suggest that even urine samples taken later in the morning are concentrated by intrinsic mechanisms, and may therefore not be representative of the overall 24 h state. Indeed, urine osmolality measured in the early or late afternoon sample was almost always (75 and 87 % of the time, respectively) within ±50 mOsm/kg of the 24 h value.

#### Saliva osmolality

Saliva osmolality has previously been shown to increase with progressive dehydration (Ely et al. 2011; Taylor et al. 2012; Walsh et al. 2004), fluid deprivation (Pross et al. 2012) and fluid restriction (Oliver et al. 2008; Pross et al. 2012). Given that an intake of 1.0 L/day is almost certainly inadequate to compensate for even minimal estimated daily water losses (EFSA 2011; IOM 2004; Sawka et al. 2005), it was hypothesized that saliva osmolality would be different between low and high fluid intakes. However, we found no difference in saliva osmolality between groups at either water intake level. Saliva osmolality was highly variable between subjects, consistent with previous results (Ely et al. 2011; Walsh et al. 2004). The broad range of saliva osmolality values in the current study is particularly interesting given that our subjects were consuming daily water volumes that fell well within the typical daily consumption range observed at the population level (IOM 2004), and were prevented from exercising, thereby minimizing sweat losses. Moreover, as seen in Fig. 4, distinct drops on the order of 10 mOsm/kg were recorded in the samples taken approximately 30 min after finishing breakfast, lunch, and afternoon snack. Of note, the postprandial drop in saliva osmolality was similar whether 0.25 or 0.5 L of water was consumed with the meal. This expands previous work (Ely et al. 2011), which reported that a water mouth rinse temporarily depressed saliva osmolality that recovered to pre-rinse levels within 15 min. In contrast, our data show a significant effect for at least 30 min after finishing a meal. It is unclear whether the depression in saliva osmolality was due to the food or the water ingested. Regardless, the data suggest a clear, but temporary depressive effect of ingesting food or beverage on saliva osmolality.

A degree of caution should be exerted when extrapolating these findings to the broader population. Participants drank only water during the study, which does not accurately reflect beverage selection in free-living conditions. Moreover, intake volume was more or less equally divided through the morning, afternoon, and evening intervals, which may not be representative of real-life consumption patterns that may influence diuretic activity (Jones et al. 2010). Nonetheless, this study provides insight into intrinsic regulatory patterns that regulate urine production and directly influence markers of urine concentration. In conclusion, urinary hydration biomarkers, but not plasma or saliva osmolality, reflect differences in daily water intake in average adults not exposed to strenuous exercise or heat. Values for urine volume, osmolality, USG and color were stable within 24 h of initiating the change in water intake. Urine samples collected during the afternoon may be particularly well-suited to replace time-consuming 24 h urine collections.

## Abbreviations

POsm: Plasma osmolality
SOsm: Saliva osmolality
UCol: Urine color
UOsm: Urine osmolality
USG: Urine specific gravity
UVol: Urine volume

## References

[CR1] Armstrong LE (2007). Assessing hydration status: the elusive gold standard. J Am Coll Nutr.

[CR2] Armstrong LE, Maresh CM, Castellani JW, Bergeron MF, Kenefick RW, LaGasse KE, Riebe D (1994). Urinary indices of hydration status. Int J Sport Nutr.

[CR3] Armstrong LE, Soto JA, Hacker FT, Casa DJ, Kavouras SA, Maresh CM (1998). Urinary indices during dehydration, exercise, and rehydration. Int J Sport Nutr.

[CR4] Armstrong LE, Pumerantz AC, Fiala KA, Roti MW, Kavouras SA, Casa DJ, Maresh CM (2010). Human hydration indices: acute and longitudinal reference values. Int J Sport Nutr Exerc Metab.

[CR5] Armstrong LE, Johnson EC, Munoz CX, Swokla B, Le Bellego L, Jimenez L, Casa DJ, Maresh CM (2012). Hydration biomarkers and dietary fluid consumption of women. J Acad Nutr Diet.

[CR6] Cheuvront SN, Fraser CG, Kenefick RW, Ely BR, Sawka MN (2011). Reference change values for monitoring dehydration. Clin Chem Lab Med.

[CR7] Clark WF, Sontrop JM, Macnab JJ, Suri RS, Moist L, Salvadori M, Garg AX (2011). Urine volume and change in estimated GFR in a community-based cohort study. Clin J Am Soc Nephrol.

[CR8] Curhan GC, Willett WC, Knight EL, Stampfer MJ (2004). Dietary factors and the risk of incident kidney stones in younger women: Nurses’ Health Study II. Arch Intern Med.

[CR9] EFSA (2011). Scientific opinion on dietary reference values for water. EFSA J.

[CR10] Ely BR, Cheuvront SN, Kenefick RW, Sawka MN (2011). Limitations of salivary osmolality as a marker of hydration status. Med Sci Sports Exerc.

[CR11] Francesconi RP, Sawka MN, Pandolf KB, Hubbard RW, Young AJ, Muza S (1985). Plasma hormonal responses at graded hypohydration levels during exercise-heat stress. J Appl Physiol.

[CR12] George CP, Messerli FH, Genest J, Nowaczynski W, Boucher R, Kuchel Orofo-Oftega M (1975). Diurnal variation of plasma vasopressin in man. J Clin Endocrinol Metab.

[CR13] Hennequin C, Daudon M, Phung T, Lacour B, Jungers P (1995). Evaluation of the lithogenic risk in renal lithiasis. Value of urine density measurement. Presse Med.

[CR14] IOM (2004). Dietary reference intakes for water, potassium, sodium, chloride, and sulfate.

[CR15] Jones EJ, Bishop PA, Green JM, Richardson MT (2010). Effects of metered versus bolus water consumption on urine production and rehydration. Int J Sport Nutr Exerc Metab.

[CR16] Kavouras SA (2002). Assessing hydration status. Curr Opin Clin Nutr Metab Care.

[CR17] Oliver SJ, Laing SJ, Wilson S, Bilzon JL, Walsh NP (2008). Saliva indices track hypohydration during 48 h of fluid restriction or combined fluid and energy restriction. Arch Oral Biol.

[CR18] Peronnet F, Mignault D, du SP, Vergne S, Le BL, Jimenez L, Rabasa-Lhoret R (2012). Pharmacokinetic analysis of absorption, distribution and disappearance of ingested water labeled with D(2)O in humans. Eur J Appl Physiol.

[CR19] Perrier E, Vergne S, Klein A, Poupin M, Rondeau P, Le BL, Armstrong LE, Lang F, Stookey J, Tack I (2012). Hydration biomarkers in free-living adults with different levels of habitual fluid consumption. Br J Nutr.

[CR20] Pross N, Demazieres A, Girard N, Barnouin R, Santoro F, Chevillotte E, Klein A, Le BL (2012). Influence of progressive fluid restriction on mood and physiological markers of dehydration in women. Br J Nutr.

[CR21] Roussel R, Fezeu L, Bouby N, Balkau B, Lantieri O, Alhenc-Gelas F, Marre M, Bankir L (2011). Low water intake and risk for new-onset hyperglycemia. Diabetes Care.

[CR22] Sawka MN, Cheuvront SN, Carter R (2005). Human water needs. Nutr Rev.

[CR23] Sorensen MD, Kahn AJ, Reiner AP, Tseng TY, Shikany JM, Wallace RB, Chi T, Wactawski-Wende J, Jackson RD, O’Sullivan MJ, Sadetsky N, Stoller ML (2012). Impact of nutritional factors on incident kidney stone formation: a report from the WHI OS. J Urol.

[CR24] Strippoli GF, Craig JC, Rochtchina E, Flood VM, Wang JJ, Mitchell P (2011). Fluid and nutrient intake and risk of chronic kidney disease. Nephrology.

[CR25] Taylor NA, van den Heuvel AM, Kerry P, McGhee S, Peoples GE, Brown MA, Patterson MJ (2012). Observations on saliva osmolality during progressive dehydration and partial rehydration. Eur J Appl Physiol.

[CR26] Walsh NP, Laing SJ, Oliver SJ, Montague JC, Walters R, Bilzon JL (2004). Saliva parameters as potential indices of hydration status during acute dehydration. Med Sci Sports Exerc.

[CR27] Watson PE, Watson ID, Batt RD (1980). Total body water volumes for adult males and females estimated from simple anthropometric measurements. Am J Clin Nutr.

